# Aggression to Biomembranes by Hydrophobic Tail Chains under the Stimulus of Reductant

**DOI:** 10.3390/molecules29174001

**Published:** 2024-08-24

**Authors:** Sijia Wang, Huifang Xu, Yuanyuan Li, Lingyi Zhang, Shouhong Xu

**Affiliations:** 1 College of Pharmacy, Henan University of Chinese Medicine, Zhengzhou 450046, China; hfxu@hactcm.edu.cn (H.X.); liyy2018@hactcm.edu.cn (Y.L.); zly01001@163.com (L.Z.); 2 Key Laboratory for Advanced Materials, School of Chemistry and Molecular Engineering, East China University of Science and Technology, Shanghai 200237, China

**Keywords:** redox responsive, viologen derivative, hydrophobic tail chain, interaction, antitumor activity

## Abstract

Stimulus-responsive materials hold significant promise for antitumor applications due to their variable structures and physical properties. In this paper, a series of peptides with a responsive viologen derivative, Pep-C_n_V (*n* = 1, 2, 3) were designed and synthesized. The process and mechanism of the interaction were studied and discussed. An ultraviolet–visible (UV) spectrophotometer and fluorescence spectrophotometer were used to study their redox responsiveness. Additionally, their secondary structures were measured by Circular Dichroism (CD) in the presence or absence of the reductant, Na_2_SO_3_. DPPC and DPPG liposomes were prepared to mimic normal and tumor cell membranes. The interaction between Pep-C_n_V and biomembranes was investigated by the measurements of surface tension and cargo leakage. Results proved Pep-C_n_V was more likely to interact with the DPPG liposome and destroy its biomembrane under the stimulus of the reductant. And the destruction increased with the length of the hydrophobic tail chain. Pep-C_n_V showed its potential as an intelligent antitumor agent.

## 1. Introduction

Antimicrobial peptides (AMPs) are a kind of short peptide with the ability of killing bacteria [1,2,3]. These peptides achieve their function by forming pores in the cell membrane, which leads to leakage of entocytes and further cell death [4,5]. This mechanism overcomes the resistance of traditional drugs and also possesses the antitumor function. AMPs have become the focus of attention in biomedicine. Up to now, over 2600 AMPs have been cataloged in the antimicrobial peptide database, and several have been used in clinics [6].

However, natural antimicrobial peptides have certain drawbacks, including susceptibility to enzymolysis and low specificity, which restrict their applications [7,8]. Researchers started developing synthetic antimicrobial peptides by modifying natural ones or designing them independently. Based on their action mechanism, the basic structural characteristics of AMPs were summarized as electropositivity and hydrophobicity [9,10,11]. Therefore, researchers designed and synthesized antimicrobial peptides utilizing these structural features. In 2019, researchers at the University of British Columbia synthesized a series of Innate Defense Regulator (IDR) peptides with enhanced properties. One such peptide, IDR-1018, was found to have broad-spectrum antimicrobial activity, including multi-drug-resistant bacteria [12]. A magainin analog, Pexiganan, was developed and tested in two Phase III clinical trials by a company called Dipexium Pharmaceuticals for the treatment of mild infections of diabetic foot ulcers [13]. It was synthesized as a topical cream and showed promising results against both Gram-positive and Gram-negative bacteria.

Look at these antimicrobial peptides; hydrophobicity is the common and important feature to realize their antimicrobial/antitumor functions. It determines AMPs’ destruction of abnormal cells. The high hydrophobicity is useful especially for the penetration in the biological membranes, which leads to the leakage of intracellular contents and ultimate cell death [9]. However, it raised another question. Exposed hydrophobic tail chains would produce non-specific toxicity to normal cells [14,15]. How to reduce the toxicity to normal cells and increase the toxicity to tumor cells?

In solid tumors, the complex tumor micro-environment was characterized by acidity [16], hyperthermia [17], low oxygen concentration [18], high reductant [19], and elevated H_2_O_2_ [20]. Based on this, environmentally responsive materials became our focus [21,22]. Shan et al. synthesized a series of pH-responsive antimicrobial peptides equipped with efficient bacterial killing activity at pH 6.5 and inactivity at pH 7.4 [23]. Li et al. developed a prototype antimicrobial peptide capable of achieving high activity exclusively at low environmental pH to target bacterial species like Streptococcus mutans [24]. Redox responsiveness was also a common method. And viologen is a well-known reduction material. It has been explored for various applications due to its unique redox properties. For instance, viologen-based materials can be engineered to release therapeutic agents in response to specific redox conditions [25]. Viologens are also employed in the development of biosensors due to their electron transfer capabilities and sensitivity to changes in the redox environment [26]. The conversion between hydrophilicity and hydrophobicity under the stimulus of Na_2_SO_3_ is crucial for realizing its antitumor potential, which will enhance its safety in clinical applications [27,28]. Based on the above, viologen was used in our study and played an important role.

**Scheme 1:**
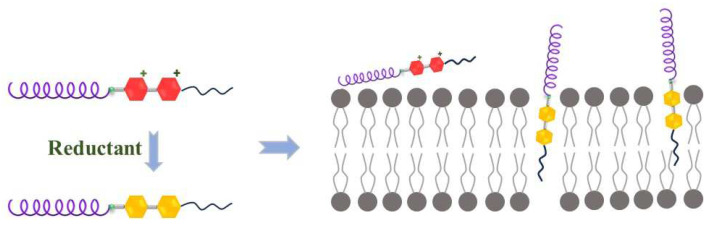
Mechanism of Pep-C_n_V with redox responsiveness.

In this work, a series of Pep-C_n_V with different alkane chain lengths were designed and synthesized, in which Pep was obtained from our previous study and held the α-helical structure [29]. As shown in Scheme 1, in the normal cellular environment, Pep-C_n_V were hydrophilic so that they were hypotoxic. Under the stimulus of the reductant, Na_2_SO_3_, viologen changed to the reduced state, which was hydrophobic, showing its aggression to biomembranes. Compared with mimic normal cells, Pep-C_n_V had better affinity for mimic tumor cells in an acidic condition, which was based on their electrostatic effect between Pep and biomembrane. And hydrophobicity became an important factor affecting Pep-C_n_V’s function. Pep-C_n_V showed their potential as antitumor agents for their aggregation/destruction to biomembranes under the stimulus of reduction. This work provided a theoretical basis for the development of synthetic AMPs.

## 2. Results and Discussions

### 2.1. Properties of Pep-C_n_V

#### 2.1.1. Fundamental Structures

**Figure 1:**
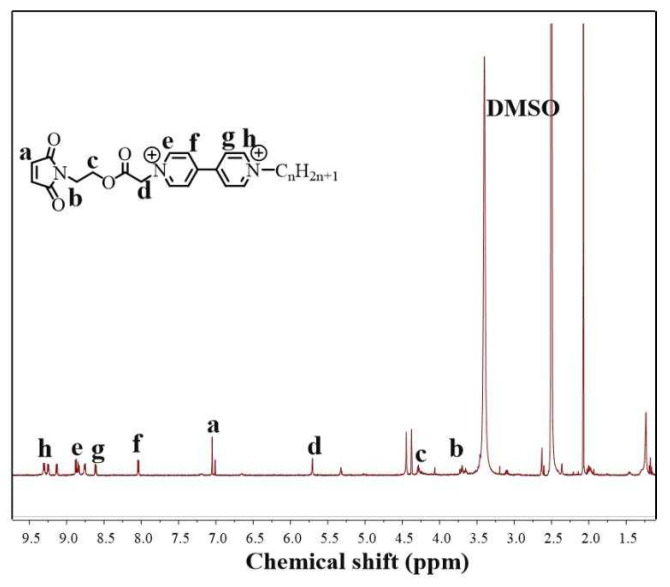
H’NMR of Mal-C_n_V.

Pep-C_n_V was obtained by chemical synthesis, and their structures were confirmed using ^1^H NMR and FTIR. As shown in Figure 1, the Mal-C_n_V was confirmed for the peaks at δ9.164 (d, 2H, Aryl-H), δ8.908 (d, 2H, Aryl-H), δ8.641 (d, 2H, Aryl-H), δ8.096 (d, 2H, Aryl-H), δ7.055 (s, 2H, -CH=CH-), δ5.724 (s, 2H, -OOCCH_2_N-), δ4.389 (s, 3H, -NCH_3_), δ 4.326 (t, 2H, -CH_2_OOC-), and δ3.699 (t, 2H, -NCH_2_-).

**Figure 2:**
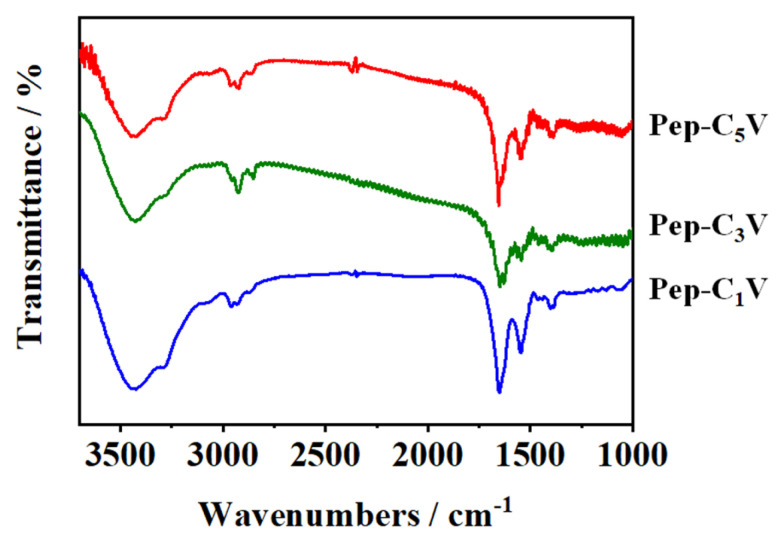
FTIR of Pep-C_n_V (*n* = 1, 3, 5).

Figure 2 shows the FTIR spectra of Pep-C_n_V. The compound Mal-C_n_V exhibited a characteristic peak of -C=C-H (3000~3100 cm^−1^) in 3037.6 cm^−1^ wavenumbers. After the sulfhydryl addition occurred between the sulfydryl group of Pep and the maleimide group of Mal-C_n_V, a new characteristic peak emerged in 2929.6 cm^−1^ wavenumbers, which can be attributed to the molecular structure of –C-C-H in the maleimide group. Thus, the results convincingly confirmed the cross-linking between the Pep and Mal-C_n_V. This result confirmed that Pep-C_n_V was successfully synthesized.

#### 2.1.2. Secondary Structures

**Figure 3:**
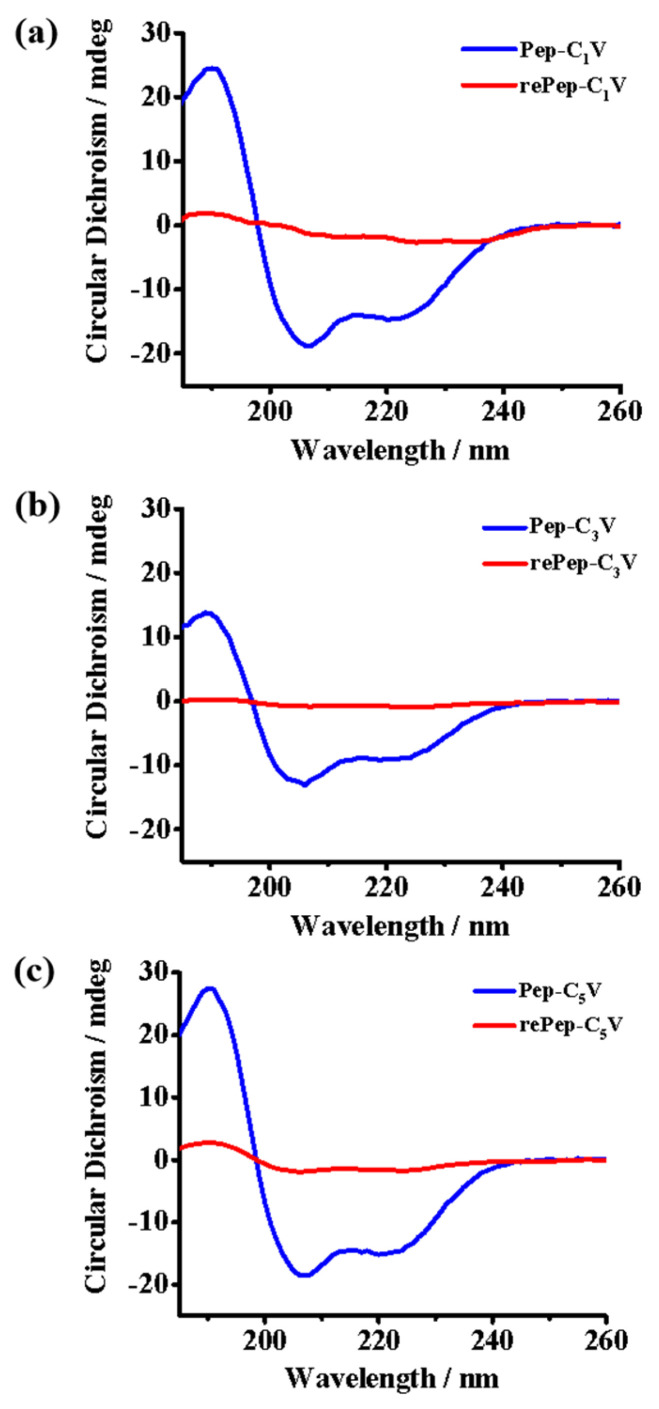
CD spectra of (**a**) Pep-C_1_V, (**b**) Pep-C_3_V and (**c**) Pep-C_5_V with reductant or not.

Peptides with α-helical structures have shown superior activities of interacting with biomembranes, which leads to disruption to the target cells. Here, the secondary structures of Pep-C_n_V with reductant or not were studied by CD spectra. As shown in Figure 3, Pep-C_n_V adopted a typical α-helix structure that possessed characteristic negative bands at 208 nm, 222 nm, and a positive band at 192 nm. On the contrary, the α-helix of reductive Pep-C_n_V (rePep-C_n_V) was weakened and gradually changed to random. Peptide’s α-helical structure was affected by hydrophobic force and electrostatic interaction [30]. Under the stimulus of Na_2_SO_3_, the state of viologen changed from oxidative V^2+^ to reductive V, accompanied by changes in secondary structures. It indicated that the reduced electrostatic interaction and enhanced hydrophobic interaction induced by C_n_V collectively influenced the secondary structure of Pep.

### 2.2. Redox Responsiveness of Pep-C_n_V

Viologens are typical redox molecules; they are positively charged and capable of being reversibly reduced to a radical cationic or neutral species by means of chemical or electrical reductions. To confirm the redox responsiveness of Pep-C_n_V, UV–vis absorption spectroscopy and fluorescence spectra were measured.

**Figure 4:**
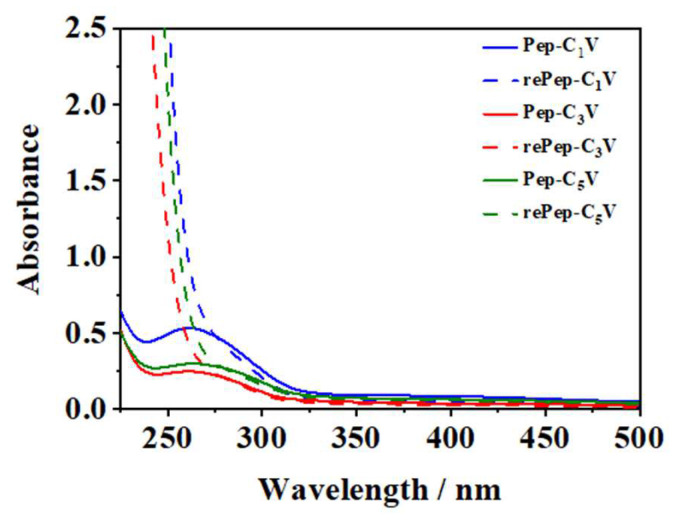
UV-vis spectra of Pep-C_n_V with reductant or not.

As shown in Figure 4, for Pep-C_n_V, their UV–vis absorption peaks were at around 260 nm. Under the stimulus of a reductant, the absorption peak of viologen (260 nm) disappeared, indicating the presence of reduced viologen. It could be considered that the hydrophilic cationic V^2+^ had turned into hydrophobic uncharged V triggered by Na_2_S_2_O_3_.

**Figure 5:**
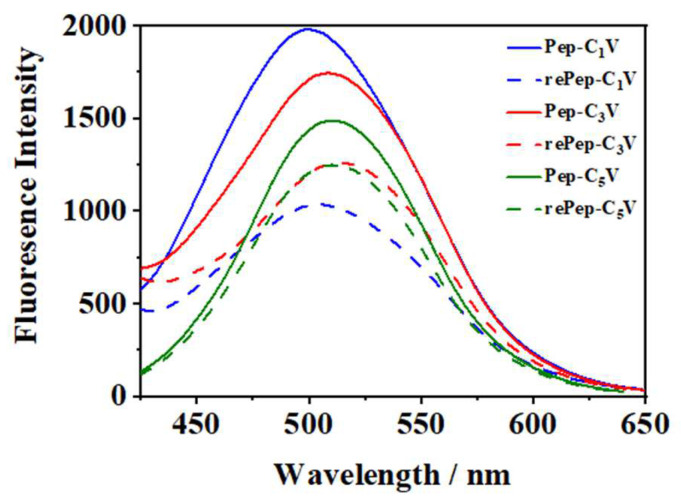
Fluorescence spectra of Pep-C_n_V with reductant or not.

Fluorescence spectra were also measured to further confirm the redox responsiveness in Figure 5. Pep-C_n_V showed strong fluorescence intensity at the wavelength of 510 nm. Upon adding the Na_2_S_2_O_3_, the fluorescence intensity was quenched significantly, owing to the change in viologens from V^2+^ to V. Pep-C_n_V showed their great redox responsiveness under the action of Na_2_S_2_O_3_.

### 2.3. Interaction between Pep-C_n_V and Biomembranes

#### 2.3.1. Surface Tension

**Figure 6:**
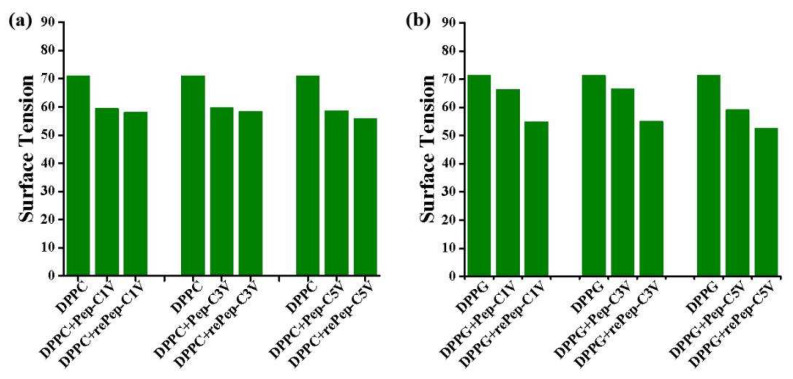
Surface tension of (**a**) DPPC and (**b**) DPPG liposomes under the interaction of Pep-C_n_V with reductant or not.

To investigate the interaction between Pep-C_n_V and biomembranes, surface tensions of liposome solutions were measured. Here, negatively charged DPPG liposomes were used as mimic tumor cells, and neutral DPPC liposomes were used as normal cells. We investigated the interaction between Pep-C_n_V and DPPC/DPPG liposomes with reductant or not at 25 °C, as shown in Figure 6.

For the DPPC liposome (Figure 6a), the addition of Pep-C_n_V reduced its surface tension. However, the presence of Na_2_S_2_O_3_ almost had no effect on surface tension. It indicated that the change in surface tension of the DPPC liposome was not induced by viologen. Surface tension could be affected by ions in the previous reports [31,32]. The significant reduction in surface tension was induced by Pep-C_n_V’s surface charge, and the slight change was induced by hydrophobic uncharged V under the stimulus of a reductant.

For DPPG liposomes (Figure 6b), the addition of Pep-C_n_V also reduced the surface tension. Different from DPPC liposomes, the rePep-C_n_V played an important role in further reducing the surface tension, confirming the function of viologen. The change in surface tension could be owed to the display of hydrophobicity. Under the stimulus of a reductant, driven by hydrophobic forces, the reduced viologens would insert into or even form pores in the membrane of the DPPG liposome. Studies have shown that the interfacial freedom of liposome membranes affects the surface tension of liposome solutions [33,34]. The inserted rePep-C_n_V reduced the interfacial freedom of the liposome membrane, resulting in a reduction in the surface tension of the solution. This result proved Pep-C_n_V had better affinity to mimic tumor cells under the stimulus of reduction. And this affinity was expected to be a potential antitumor agent.

#### 2.3.2. Cargo Leakage

**Figure 7:**
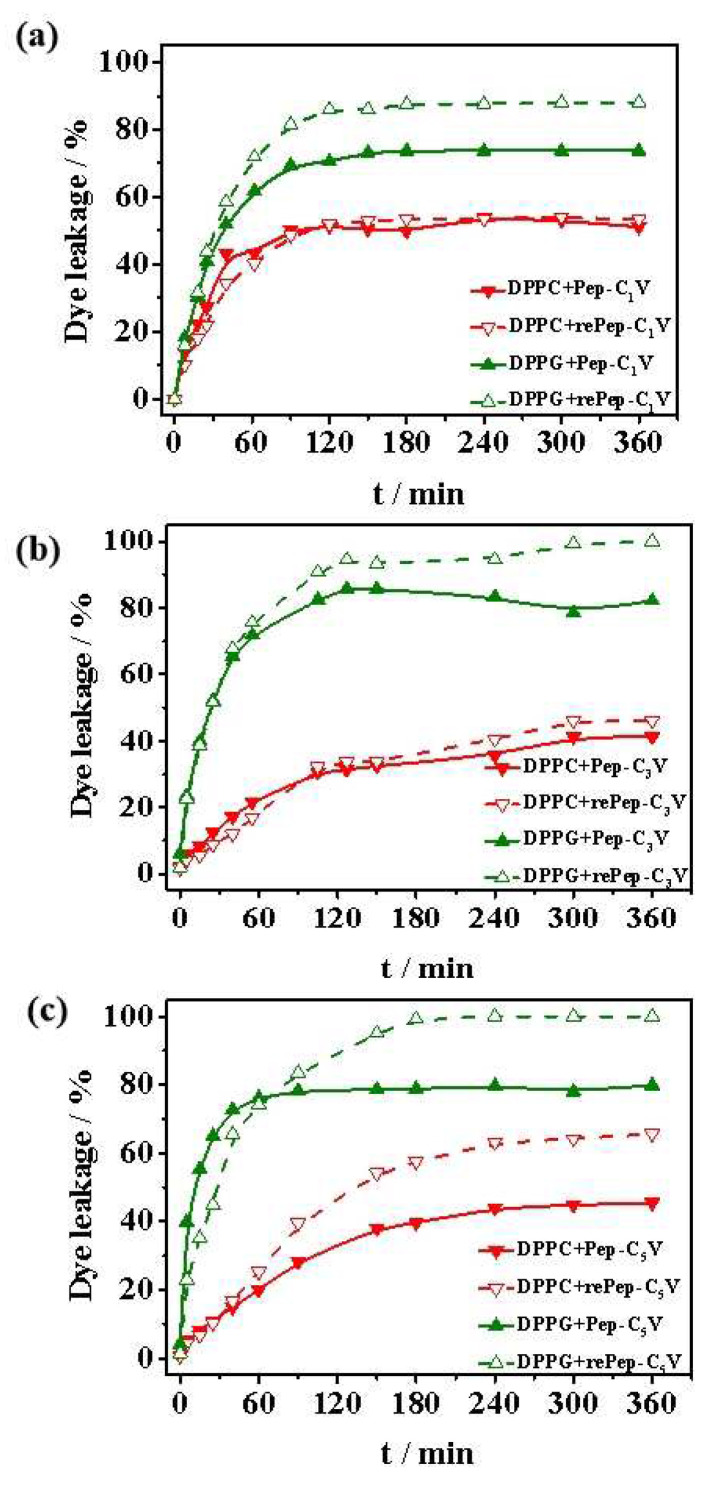
Cargo leakage from DPPC and DPPG liposomes induced by (**a**) Pep-C_1_V, (**b**) Pep-C_3_V and (**c**) Pep-C_5_V with reductant or not.

Rhodamine 6G was loaded into liposomes to mimic the cytoplasm, and its leakage kinetics incubating with Pep-C_n_V under the stimulus of reductant or not were investigated for discussing their aggression to biomembranes in Figure 7.

Firstly, biocompatibility was investigated. For DPPC liposomes, the cargo leakages induced by Pep-C_n_V were all less than these from DPPG liposomes. And the reductant did not have much effect on cargo leakages for rePep-C_1_V and rePep-C_3_V. For Pep-C_5_V, the stimulus of reductant improved the cargo leakage for its longer hydrophobic tails. This result proved Pep-C_n_V almost had no destructive effect on DPPC liposomes. It exhibited some cytotoxicity only when the hydrophobicity was strong and the hydrophobic tail chain was exposed. This result proved exposed hydrophobic tail chains would produce non-specific toxicity to normal cells.

Then, aggression to biomembranes was studied. For DPPG liposomes, the behavior of cargo leakages was different. The cargo leakages were all improved with the addition of Pep-C_n_V. It proved the interaction between Pep-C_n_V and DPPG liposomes was stronger, owing to the electrostatic interaction between them. Then, the cumulative leakages reached the maximum under the stimulus of reductant, indicating the insertion of hydrophobic reduced C_n_V. This result was consistent with the results of surface tension in Section 2.3.1.

Finally, the destruction of Pep-C_n_V to biomembranes with hydrophobic tails of different lengths was compared. In the reduced states, the cargo leakage from DPPG liposomes induced by rePep-C_3_V and rePep-C_5_V reached almost 100%. It was 87.1% for rePep-C_1_V. The function of rePep-C_n_V increased with the increase in hydrophobic tails, proving the role of hydrophobicity in realizing tumor cells’ destruction. The stronger the hydrophobicity, the stronger the effect on the biomembranes. Combined with the above biocompatibility studies, Pep-C_3_V possessed significant advantages with biocompatibility for normal cells and destructiveness for tumor cells. Pep-C_n_V would be a potential antitumor agent under the stimulus of a reductant.

**Scheme 2:**
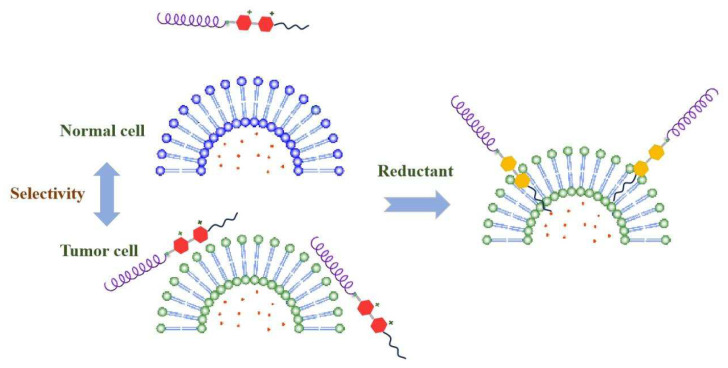
Pep-C_n_V’s selectivity and destructiveness for DPPG liposomes under the stimulus of reductant.

Scheme 2 concluded the process of interaction between Pep-C_n_V and biomembranes. On the basis of electrostatic interaction, Pep-C_n_V absorbed more on the surface of DPPG liposomes, proving its potential selectivity of tumor cells. Then, under the stimulus of reductant, the hydrophilic V^2+^ changed to the hydrophobic molecule V. Driven by hydrophobic forces, the adsorbed Pep-C_n_V further inserted into the biomembrane, inducing cell death. And the stronger the hydrophobicity, the stronger the effect on the biomembranes. Pep-C_n_V showed its potential as a novel antitumor agent.

## 3. Materials and Methods

### 3.1. Materials and Reagents

Peptides with the sequence of NH_2_-C-VAQLEVK-VAQLESK-VSKLESK-VSSLESK-COOH, named Pep, were synthesized by Top-Peptide Co., Ltd. (purity > 95%) (Shanghai, China). Furthermore, 4,4-bipyridine and relevant alkyl halide were obtained from J&K Scientific (purity > 98%) (Beijing, China). Moreover, 2-bromoisobutyryl bromide and N-(2-hydroxyethyl) maleimide were obtained from Aladdin Industrial Corporation (purity > 98%) (Shanghai, China); 1,2-dipalmitoyl-sn-glycerol-3-phosphocholine (DPPC, purity > 98%) and 1,2-dipalmitoyl-sn-glycerol-3-phospho-(1-rac-glyerol) (DPPG, purity > 98%) were obtained from Lipoid (Ludwigshafen, Germany); Rhodamine 6G was purchased from J&K Scientific (Beijing, China); Sephadex G-25 was purchased from Shanghai Haoran Biological Technology Co. Ltd. (Shanghai, China). Other chemical reagents and solvents were of analytical grade and obtained from commercial suppliers.

### 3.2. Synthesis of Pep-C_n_V

**Figure 8:**
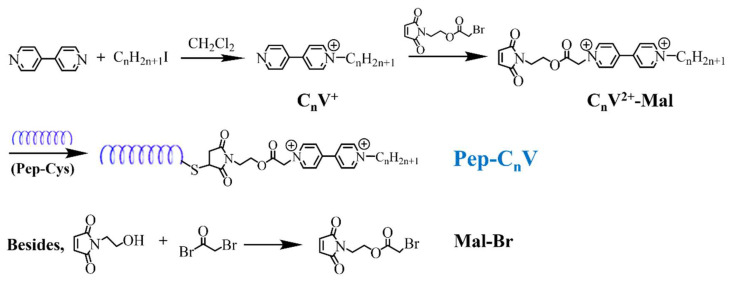
Synthetic procedures of Pep-C_n_V.

The synthesis procedures of Pep-C_n_V were performed according to the literature previously reported [35]. There were four steps from 4,4-bipyridine to Pep conjugation, as shown in Figure 8.

***Synthesis of Mal-Br***. Mal-Br was obtained from the reaction between N-(2-hydroxyethyl) maleimide and trimethylamine (molar ratio: 1/1.5) in CH_2_Cl_2_ solution under an ice-water bath for 30 min and then at room temperature for another 12 h. Then, after processing extraction, concentration, and drying, Mal-Br was obtained.

***Synthesis of C_n_V^+^***. C_n_V^+^ was obtained from the reaction between 4,4-bipyridine and alkyl halide in the inert condition, avoiding light. Solvents and reaction temperatures were different for the different reactivity of alkyl halides. When n = 1, the reaction condition was room temperature for 24–48 h with light avoidance, and the reaction solvent was dichloromethane. When n = 3, the condition remained unchanged. When n = 5, the reaction condition became more stringent with the temperature of 70–80 °C, and the reaction solvent changed to anhydrous acetonitrile.

***Synthesis of C_n_V^2+^-Mal***. C_n_V^+^ and Mal-Br (molar ratio: 1/1.2) were mixed in anhydrous acetonitrile and refluxed at 90 °C for 72 h. Then, the solid precipitated and was filtered/washed three times with acetonitrile. Then, the precipitation was dried in a vacuum oven to obtain C_n_V^2+^-Mal.

***Conjugation of C_n_V^2+^-Mal to Pep***. The conjugation was achieved by the reaction between the thiol group at the C-terminus of Pep and the maleimide group of C_n_V^2+^-Mal. Because of the water solubility of Pep and C_n_V^2+^-Mal, the reaction was in Tris–HCl buffer at 25 °C for 12 h. After the reaction was completed, the mixture was put into the dialysis bag (MWCO:3500), dialyzing for 48 h. The dialysate was changed every 6 h to remove unbound peptides. Finally, Pep-C_n_V were obtained.

^1^H NMR (AVANCE III 400, Brucker, Leipzig, Germany) and FT-IR (Nicolet 5700, Thermo Fisher, Waltham, MA, USA) were used to characterize the structure of the above products.

### 3.3. Secondary Structure Studies

To analyze the secondary structure of Pep-C_n_V, Circular Dichroism (CD) scans were applied using a Circular Dichroism Spectrometer (Chirascan, Applied Photophysics, Leatherhead, UK). A certain amount of Pep-C_n_V was dissolved in the deionized water. Then, the CD spectrum was obtained with reductant or not over a wavelength range of 190 to 260 nm at the speed of 1 nm/s and bandwidth of 1.0 nm.

### 3.4. Responsiveness Studies

Redox responsiveness of Pep-C_n_V was studied by UV spectrophotometer (UV-2450, Shimadzu, Kyoto, Japan) and fluorescence spectrophotometer (F-4500, Hitachi, Tokyo, Japan). A UV spectrophotometer was recorded with reductant or not, and Pep-C_n_V’s redox responsiveness was judged by comparing the positions of the peaks. Similarly, the fluorescence intensity (λ_ex_ = 360 nm, λ_em_ = 490 nm, 10 nm slits) were also measured under the action of reductant or not. A comparison of the intensity of peaks was used to confirm its redox responsiveness.

### 3.5. Construction of Cell Models

The cytoplasmic membrane of normal mammals is well known to contain several kinds of lipids containing phosphatidylcholine (PC), sphingomyelin, and cholesterol. PC has been reported to be enriched in the outer monolayer. For tumor cells whose membrane surfaces are electronegative, phosphatidylglycerol (PG) was chosen as the abnormal cell membrane mimicking system. Hence, DPPC and DPPG were used in this study to construct a model cell membrane system. A thin film hydration method was used to prepare liposomes as normal and tumor cell membranes. DPPC and DPPG were dissolved in the methanol/chloroform mixture, and the mixtures were evaporated. Then, the lipid films were hydrated using 5 mL Tris–HCl buffer (10 mM, pH 7.4) to obtain the liposomes (2 mg/mL). To obtain liposomes with good stability and uniform size, they were extruded several times through 200 nm polycarbonate membranes by a mini-extruder (LiposoFast-Basic, Avestin, Ottawa, Canada). Sizes and zeta potentials of the liposomes were measured using dynamic light scattering (DLS) (Zetasizer Nano-ZS, Malvern, UK) at 25 °C.

### 3.6. Surface Tension Studies

Surface tension was related to the surface adsorption concentration. It was performed in this work to investigate the interaction between biomembranes and Pep-C_n_V. The surface tensions of mimic cells mixed with Pep-C_n_V were measured using a surface tensiometer (Sigma 703D, Biolin, Espoo, Finland) at 25 °C. The mixtures of mimic cells (DPPC/DPPG liposomes) and Pep-C_n_V were prepared by mixing and stirring at room temperature for 6 h with reductant or not. Then, the surface tensions were measured and compared.

### 3.7. Cargo Leakage Studies

Rhodamine 6G@liposomes were prepared in which Rhodamine 6G was chosen as the fluorescent dye to mimic the cytoplasm of cells, and its leakage behavior was assayed to investigate the interaction between Pep-C_n_V and biomembranes. Cargo leakage was performed at 37 °C by the dialysis method. The amount of Pep-C_n_V with reductant or not was mixed with Rhodamine 6G@liposome. The mixture was put into a 2000 kDa molecular weight cut-off dialysis tube and dialyzed against Tris–HCl buffer (2 Mm, pH = 7.4). At various time points, the buffer was withdrawn and measured by fluorescence spectrophotometry (F-4500, Hitachi, Japan) (λex = 525 nm, λem = 550 nm, and 10 nm of both slit width). The fluorescence intensity of the buffer at every time point was defined as *A*_t_. The initial total amount of Rhodamine 6G loaded in the liposome was defined as *A*_0_ after disrupting liposomes with 10% (*v*/*v*) Triton X-100 solution. The cumulative leakage amount of Rhodamine 6G was calculated as *A*_t_/*A*_0_.

## 4. Conclusions

In this paper, we designed and synthesized a series of peptide derivatives Pep-C_n_V with alkane chains of different lengths. Pep-C_n_V had shown their redox responsiveness under the stimulus of reductant, Na_2_S_2_O_3_. And their secondary structures changed triggered by the reductant. Surface tension studies suggested the interaction between Pep-C_n_V and biomembranes, which was mainly controlled by the hydrophobic force. Cargo leakage studies confirmed Pep-C_n_V’s function on biomembranes. Pep-C_n_V showed their good biocompatibility and held preferential destructiveness to mimic tumor cells accompanied by the addition of reductant. Preferably, Pep-C_3_V showed its potential as an antitumor agent with low side effects and high antitumor efficiency. Based on the above results, the design of Pep-C_n_V involved incorporating alkane chains of different lengths to modulate the hydrophobic interactions with biomembranes, enhancing their efficacy. Additionally, the redox-responsive nature of Pep-C_n_V allowed for function activation in the presence of reductants. This approach minimizes off-target effects and enhances efficacy. This paper not only provides a theoretical foundation for the development of novel antitumor agents but also suggests a practical approach to designing molecules with enhanced selectivity and reduced toxicity for clinical applications.

## Data Availability

Data is contained within the article.
